# The Inhibition of Aldose Reductase Accelerates Liver Regeneration through Regulating Energy Metabolism

**DOI:** 10.1155/2020/3076131

**Published:** 2020-02-27

**Authors:** Chang Xian Li, Hong Wei Wang, Wang Jie Jiang, Gao Chao Li, Yao Dong Zhang, Chen Huan Luo, Xiang Cheng Li

**Affiliations:** Hepatobiliary Center, The First Affiliated Hospital of Nanjing Medical University, Key Laboratory of Living Donor Liver Transplantation, Nanjing, Jiangsu Province, China

## Abstract

**Objectives:**

Our previous study showed that aldose reductase (AR) played key roles in fatty liver ischemia-reperfusion (IR) injury by regulating inflammatory response and energy metabolism. Here, we aim to investigate the role and mechanism of AR in the regeneration of normal and fatty livers after liver surgery.

**Methods:**

The association of AR expression with liver regeneration was studied in the rat small-for-size liver transplantation model and the mice major hepatectomy and hepatic IR injury model with or without fatty change. The direct role and mechanism of AR in liver regeneration was explored in the AR knockout mouse model.

**Results:**

Delayed regeneration was detected in fatty liver after liver surgery in both rat and mouse models. Furthermore, the expression of AR was increased in liver after liver surgery, especially in fatty liver. In a functional study, the knockout of AR promoted liver regeneration at day 2 after major hepatectomy and IR injury. Compared to wild-type groups, the expressions of cyclins were increased in normal and fatty livers of AR knockout mice. AR inhibition increased the expressions of PPAR-*α* and PPAR-*γ* in both normal liver and fatty liver groups after major hepatectomy and IR injury. In addition, the knockout of AR promoted the expressions of SDHB, AMPK, SIRT1, and PGC1-*α* and PPAR-

**Conclusions:**

The knockout of AR promoted the regeneration of normal and fatty livers through regulating energy metabolism. AR may be a new potential therapeutic target to accelerate liver regeneration after surgery.

## 1. Introduction

Hepatectomy and liver transplantation are effective treatments for all kinds of liver diseases. Nonalcoholic fatty liver disease (NAFLD) is a common cause of chronic liver disease, and its worldwide prevalence continues to increase with the growing epidemic of obesity and diabetes [1]. It is reported that more than 20% of the patients planned for liver resection have some degree of steatosis, which is associated with increased risk of postoperative complications and death [2, 3]. Furthermore, steatotic liver graft also increased the risk of primary nonfunction or dysfunction after transplantation compared to normal graft [2, 3]. Research showed that fatty liver is more vulnerable to ischemia-reperfusion (IR) injury and then impaired liver regeneration and recovery, resulting in an amplified postoperative morbidity and mortality of patients [4, 5]. Therefore, clarifying the mechanism of fatty liver regeneration after an operation and finding effective intervention methods to promote fatty liver regeneration are very important for the recovery of liver function and improvement of long-term survival.

Aldose reductase (AR), a member of the aldo-keto reductase super family, is the first enzyme in the polyol pathway and converts glucose to sorbitol in the presence of NADPH as cofactor. AR plays important roles in the pathogenesis of diabetic complications such as cataractogenesis, retinopathy, neuropathy, and cardiovascular disease [6]. The inhibition of AR has been an attractive approach for the treatment and management of diabetic complications. Furthermore, more evidence showed that AR is upregulated and plays key roles in a number of inflammatory diseases [6–8]. The inhibition of AR suppressed the activation of transcription factors NF-*κ*B and inflammatory cytokine expression in macrophages [9, 10]. AR has been reported to be involved in the development of NAFLD. AR inhibitors may improve NAFLD through attenuating oxidative stress and inflammatory cytokine expression [11]. Moreover, experimental studies have demonstrated that AR was overexpressed in ischemic myocardium and mediated myocardial IR injury by opening the mitochondrial permeability transition pore [12]. Our previous study showed that AR played key roles in hepatic IR injury [13–15]. AR was overexpressed in ischemic liver, especially in fatty liver, and its deficiency attenuated hepatic IR injury in both normal and fatty livers by reducing liver inflammatory responses [13]. Furthermore, AR inhibition also attenuated steatotic liver injury by maintaining the homeostasis of NAD(P)(H) contents and regulating energy metabolism [14, 15]. Recently, research showed that the inhibition of AR enhanced lens regeneration by regulating the response of lens epithelial cells [16]. However, the role and mechanism of AR in the regeneration of normal liver and fatty liver after liver surgery are still unknown.

In this study, we aimed to explore the effect and mechanism of AR in the regeneration of normal and fatty livers. Firstly, the association of AR expression with liver regeneration was studied in the rat liver transplantation model and in the mice model with or without fatty change. The direct role of AR in liver regeneration was explored in the AR knockout mouse major hepatectomy and hepatic IR injury model. Together, our study found that the inhibition of AR accelerated the regeneration of normal and fatty livers through regulating energy metabolism.

## 2. Materials and Methods

### 2.1. Animal Models

Male Sprague-Dawley rats (6-8 weeks) were used as donors and recipients. C57BL/6 (6-8 weeks) mice and AR knockout mice were applied in the mouse model. Fatty livers of rats and mice were induced by feeding with a high-fat diet (TestDiet, USA) for 2 weeks. Animals were allowed free access to food and water in a room with a 12-hour light, 12-hour dark cycle. The experimental protocol was approved by the Committee on the Use of Live Animals in Teaching and Research, Nanjing Medical University.

Rat orthotopic liver transplantation was established using small-for-size liver graft with or with fatty change [13]. The median lobe, right lobe, and triangle lobe of the liver were selected to be small-for-size grafts, and the median ratio of graft weight to recipient liver weight was about 50%. Rat orthotopic liver transplantation was conducted in 2 groups: (I) normal graft group and (II) fatty graft group. Liver samples were sampled at days 2, 4, 7, and 14 after transplantation for further studies. To mimic the clinical situation, AR knockout and wild-type mice models with major hepatectomy and hepatic IR injury were applied. The branches of the hepatic artery and portal vein to the right and triangle lobes were clamped for 45 minutes by microvessel clamps followed by reperfusion. Major hepatectomy of the left and caudate lobes were performed during the ischemia duration.

Liver samples and blood were collected at days 1, 2, 4, and 7 after reperfusion.

### 2.2. Immunohistochemical Staining

The expression of PCNA was detected by immunohistochemical staining (IHC). The detail of IHC staining was described in our previous paper [17].

### 2.3. Detection of Gene Expression by Real-Time RT-PCR

Each 1 *μ*g of total RNA from different samples was used to synthesize 22 *μ*l of cDNA using the High-Capacity cDNA Kit (Applied Biosystems, Foster City, CA). RT-PCR was done with a modified version of a previous method [13]. All samples were detected in triplicate, and the readings from each sample and its internal control were used to calculate the gene expression level. After normalization with the internal control, the gene expression levels were expressed as folds relative to the control liver.

### 2.4. Measurement of Protein Levels by Western Blotting

Western blotting was done with a modified version of a previous method. *β*-Actin, anti-p-AMPK, and anti-SIRT1 antibodies were purchased from Cell Signaling Technology and Abcam.

### 2.5. ATP Assay

In order to investigate the role of AR in energy metabolism, the content of ATP was detected by an ATP assay. The ATP assay (Sigma-Aldrich) was performed according to the manufacturer's instructions.

### 2.6. Statistics and Data Analyses

Continuous variables were expressed as average with standard deviation (SD). The Mann-Whitney *U* test was used for statistical comparison. Significance was defined as *P* < 0.05. Calculations were performed by using the SPSS computer software version 16. (SPSS Inc., Chicago, IL, USA).

## 3. Results

### 3.1. Regeneration of Fatty Liver Was Inhibited after Liver Surgery

**Figure 1:**
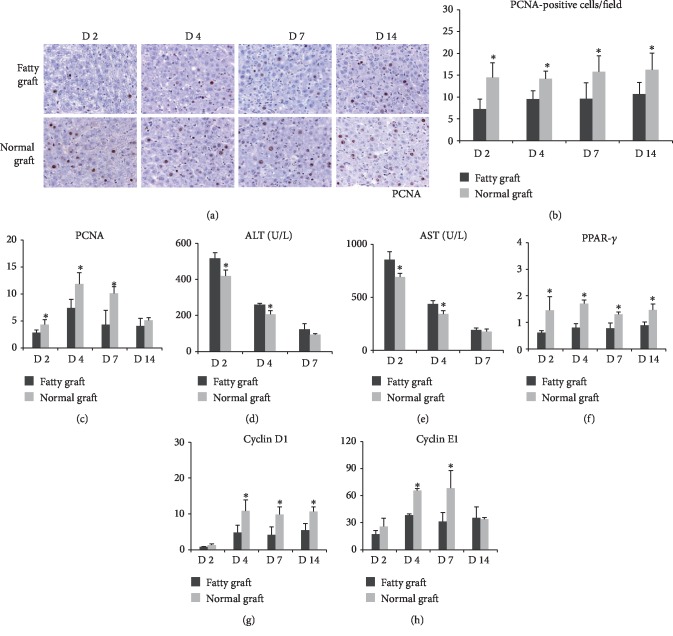
Regeneration of fatty liver was inhibited after liver surgery. (a–c) Liver regeneration was delayed in the small-for-size fatty liver graft after transplantation. (d–e) The levels of AST and ALT were increased in the small-for-size fatty graft compared with the small-for-size normal graft. (f–h) The mRNA expressions of PCNA, PPAR-*γ*, cyclin D1, and cyclin E1 in the fatty liver graft were decreased after transplantation (^∗^*P* < 0.05, *N* = 3‐6/group).

**Figure 2:**
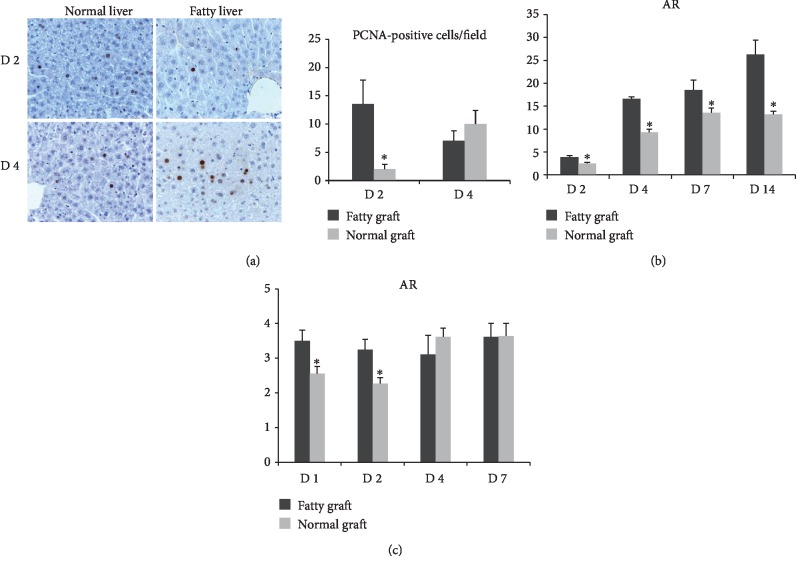
The expression of AR was upregulated in fatty liver after liver surgery. (a) Liver regeneration was delayed in mouse fatty liver after hepatectomy and IR injury. (b) The expression of AR was upregulated in the fatty liver graft after liver transplantation compared to normal liver. (c) The expression of AR was upregulated in fatty liver after hepatectomy and IR injury compared to normal liver (^∗^*P* < 0.05, *N* = 3‐6/group).

In order to investigate the effect of steatosis on liver graft regeneration after transplantation, the rat orthotopic transplantation model was established using the small-for-size fatty graft and the small-for-size normal graft. The IHC-staining data showed that hepatocyte regeneration with PCNA staining was markedly reduced in the small-for-size fatty graft compared with the small-for-size-normal graft at days 2, 4, 7, and 14 after transplantation (Figure 1(a)). The number of PCNA-positive cells were significantly lower in the small-for-size fatty graft than those in the small-for-size normal graft (Figure 1(b)). The q-PCR data also confirmed that the mRNA expression level of PCNA was decreased in the small-for-size fatty graft compared to the small-for-size normal graft (Figure 1(c)). The levels of AST and ALT were increased in the small-for-size fatty graft compared to the small-for-size normal graft (Figures 1(d) and 1(e)). Furthermore, low expressions of PPAR-*γ*, cyclin D1, and cyclin E1 were found in the small-for-size fatty graft (Figures 1(f)–1(h)). These results were also confirmed in the mouse major hepatectomy and IR injury model (Figure 2(a)). These results suggested that regeneration of fatty liver was inhibited after liver surgery.

### 3.2. AR Was Upregulated in Fatty Liver after Liver Surgery

In order to explore the mechanism of fatty liver graft delayed regeneration after surgery, we firstly detected the expression profile of genes in the liver graft after liver transplantation. The cDNA screening showed that AR was upregulated in the small-for-size fatty graft compared to the small-for-size normal graft. The real-time PCR confirmed that the expression of AR was increased in the liver graft at days 2, 4, 7, and 14 after transplantation, especially in the small-for-size fatty graft (Figure 2(b)). We further detected the expression of AR in the mouse major hepatectomy and partial I/R injury model. Similar to the results in rat transplantation, the expression of AR was higher in fatty liver after major hepatectomy and partial I/R injury compared to normal liver (Figure 2(c)).

### 3.3. The Knockout of AR Attenuated Hepatic Injury and Increased the Weight Ratio of Liver to Body after Hepatectomy and IR Injury

**Figure 3:**
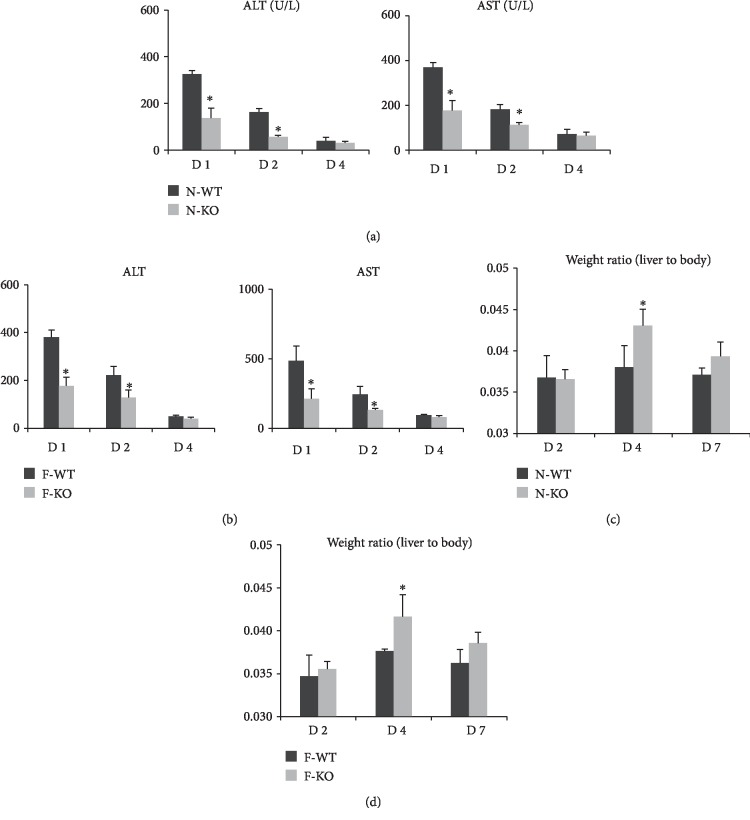
The knockout of AR attenuated hepatic injury and increased the weight ratio of liver to body after hepatectomy and IR injury. (a–b) The levels of AST and ALT were decreased in the KO groups after hepatectomy and IR injury compared to the wild-type groups. (c–d) The knockout of AR increased the weight ratio of liver to body at day 4 after hepatectomy and IR injury compared to the wild-type group (^∗^*P* < 0.05, *N* = 4‐6/group).

Our previous data showed that compared with wild-type groups, hepatic lobular architecture and portal tracts were well preserved in both the N-KO and F-KO groups after reperfusion [13]. In this study, our data showed that the levels of AST and ALT were decreased in the KO groups after hepatectomy and IR injury compared with the wild-type groups (Figures 3(a) and 3(b)). We also compared the weight ratio of liver to body at different time points after IRI. The results showed that the knockout of AR increased the weight ratio of liver to body in normal liver at day 4 after hepatectomy and IR injury (Figure 3(c)). Similar results were also found in fatty liver: a higher weight ratio of liver to body was found in the fatty KO group at day 4 after hepatectomy and IR injury compared to the wild-type fatty group (Figure 3(d)).

### 3.4. The Knockout of AR Promoted Liver Regeneration after Major Hepatectomy and IR Injury

**Figure 4:**
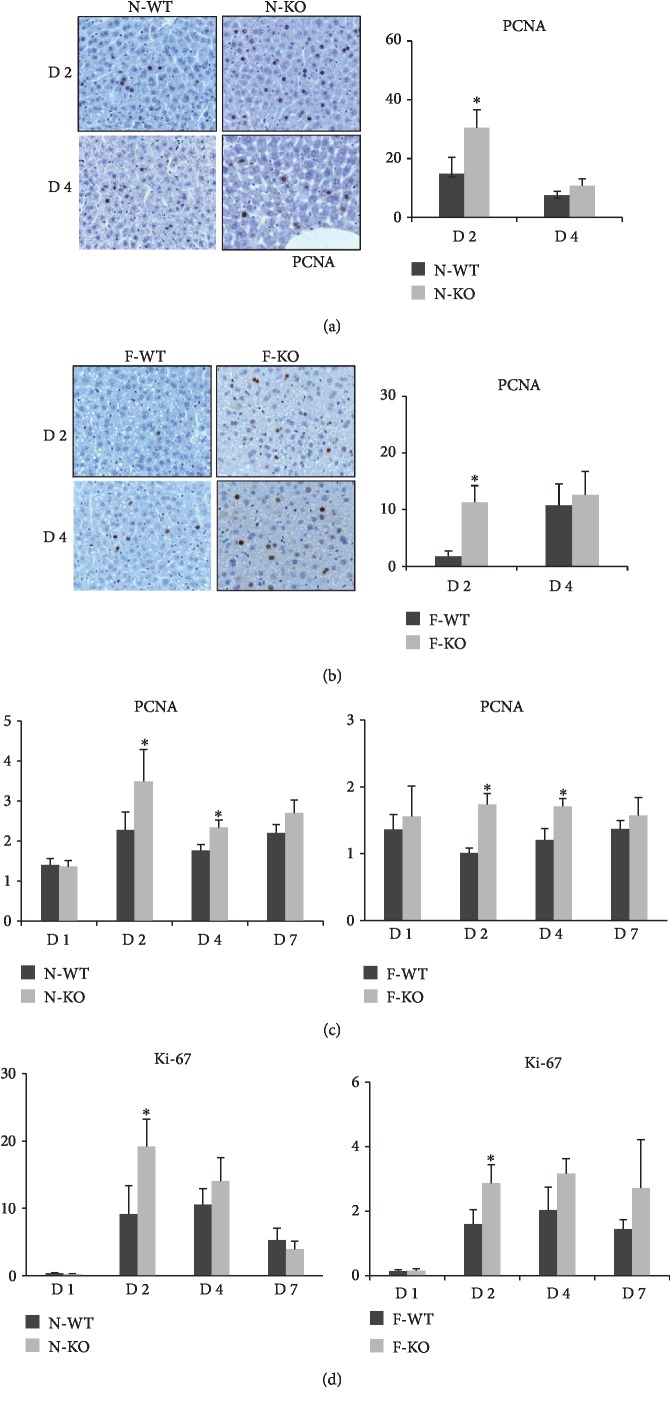
The knockout of AR accelerated liver regeneration in the mouse model. (a–b) AR deficiency significantly promoted hepatocyte regeneration with PCNA staining compared with the wild-type group at day 2 and after major hepatectomy and IR injury. (c–d) Compared to control group, the knockout of AR exhibited higher mRNA expression levels of PCNA and ki-67 in liver (^∗^*P* < 0.05, *N* = 4‐6/group).

In order to explore the role of AR in liver regeneration, the mouse major hepatectomy and IR injury model was established in AR knockout or wild-type mice with or without fatty change. In normal liver groups, our results showed that AR deficiency significantly promoted hepatocyte regeneration with PCNA staining compared with the wild-type group at day 2 after major hepatectomy and IR injury (Figure 4(a)). Compared to the wild-type group, the knockout of AR exhibited more PCNA-positive nuclei and higher mRNA expression levels of PCNA and ki-67 in normal liver (Figures 4(a)–4(d)). A similar result was also found in the fatty liver group: more PCNA-positive cells and higher mRNA expression levels of PCNA and ki-67 were found in knockout mice (Figures 4(b)–4(d)). These data indicated that the knockout of AR promoted liver generation after major hepatectomy and IR injury in both normal liver and fatty liver.

### 3.5. The Knockout of AR Increased the Cyclin Expressions in Liver after Major Hepatectomy and IR Injury

**Figure 5:**
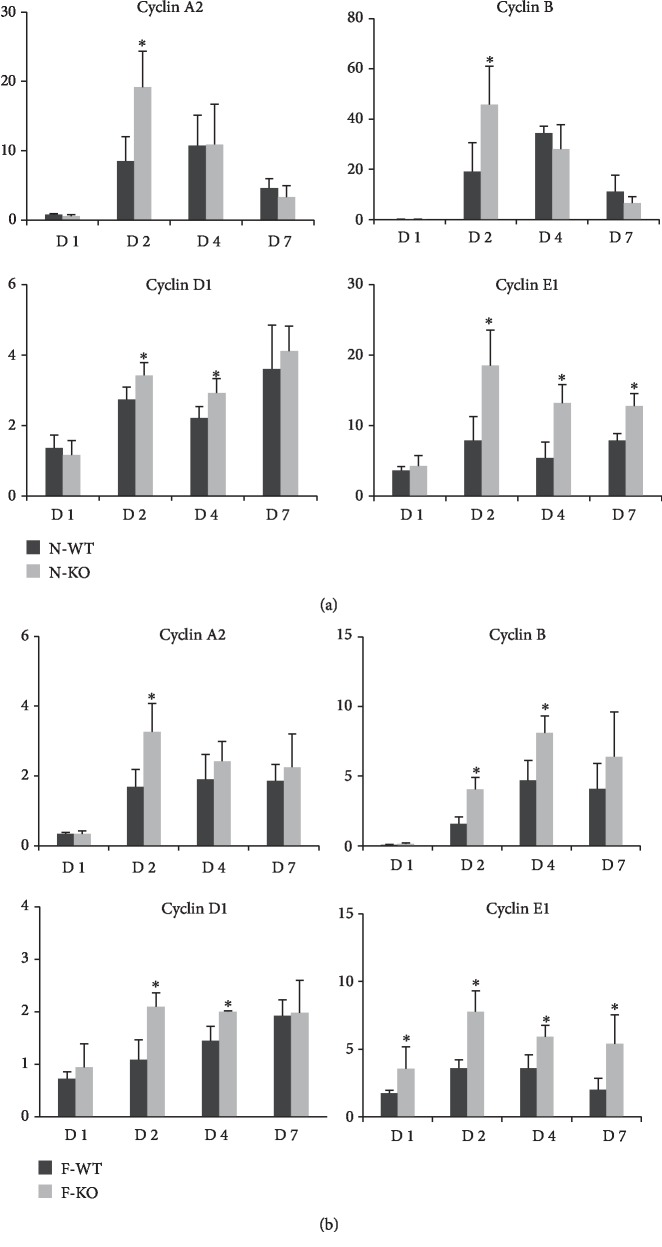
The knockout of AR increased the cyclin expressions in liver after major hepatectomy and IR injury. (a) The expressions of cyclin A2, B, D1, and E1 in normal liver were found to be increased in the N-KO group after major hepatectomy and IR injury. (b) The knockout of AR increased the mRNA levels of cyclin A2, B, D1, and E1 in fatty liver after major hepatectomy and IR injury (^∗^*P* < 0.05, *N* = 4‐6/group).

In order to further explore the mechanism of AR in liver regeneration, we detected the expressions of several cyclins such as cyclin A2, B, D1, and E1. Cyclins are important downstream effectors of diverse proliferative and transforming signaling pathways. In the normal liver group, the expressions of cyclin A2, B, D1, and E1 in liver were found to be more elevated in the N-KO group after major hepatectomy and IR injury compared to the N-WT group (Figure 5(a)). Consistent with the results of normal livers, the knockout of AR also upregulated the mRNA levels of cyclin A2, B, D1, and E1 in fatty liver after major hepatectomy and IR injury compared with the F-WT group (Figure 5(b)).

### 3.6. The Knockout of AR Promoted Mitochondrial Biogenesis and Energy Metabolism in Liver after Major Hepatectomy and IR Injury

**Figure 6:**
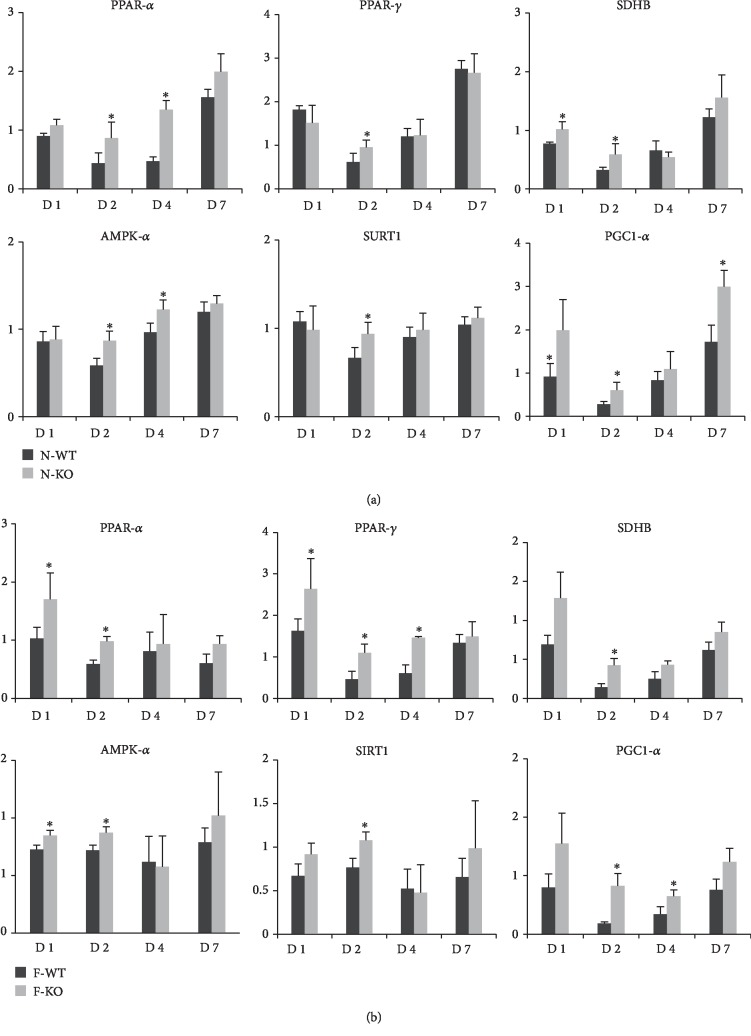
The knockout of AR promoted mitochondrial biogenesis and energy metabolism in liver after major hepatectomy and IR injury. (a) The expressions of PPAR-*α*, PPAR-*β*, SDHB, AMPK, SIRT1, and PGC1 in normal liver were found to be increased in the N-KO group after major hepatectomy and IR injury. (b) The knockout of AR increased the mRNA levels of PPAR-*α*, PPAR-*β*, SDHB, AMPK, SIRT1, and PGC1 in fatty liver after major hepatectomy and IR injury (^∗^*P* < 0.05, *N* = 4‐6/group).

PPARs (peroxisome-proliferator-activated receptors) represent a group of nuclear receptors that plays pivotal roles in the regulation of energy metabolism. Our research showed that the knockout of AR increased the expression of PPAR-*α* in both the normal liver and fatty liver groups after major hepatectomy and IR injury compared to that of the wild-type groups (Figures 6(a) and 6(b)). Furthermore, the expression of PPAR-*γ* was higher in the N-KO and F-KO groups than those in the corresponding wild-type groups (Figures 6(a) and 6(b)).

**Figure 7:**
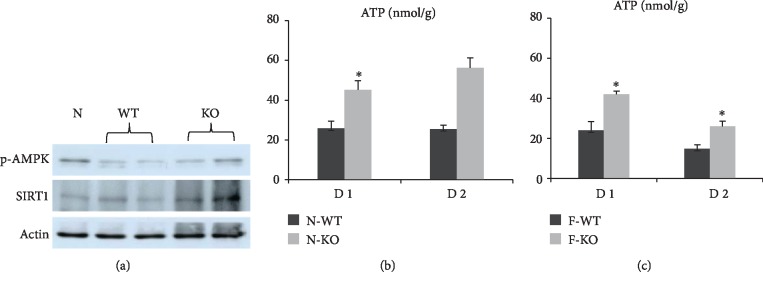
The knockout of AR increased ATP content in liver after major hepatectomy and IR injury. (a) The knockout of AR increased the expression of p-AMPK and SIRT1 in liver after major hepatectomy and IR injury compared to the wild-type group. (b) The knockout of AR increased the content of ATP in both normal and fatty livers after major hepatectomy and IR injury compared to the wild-type group (^∗^*P* < 0.05, *N* = 4‐6/group).

Mitochondria are important organelles for the energy metabolism of hepatocytes, while AMPK signaling is a key regulator of bioenergy metabolism. To further investigate the effect of AR in AMPK signaling during liver regeneration, the expressions of AMPK signaling such as AMPK, SIRT1, and PGC1-*α* in liver were detected by q-PCR. In normal liver, the mRNA expressions of AMPK, SIRT1, and PGC1-*α* were increased in AR knockout mice compared to wild-type mice (Figure 6(a)). The western blot data also confirmed that the knockout of AR increased the expression of p-AMPK and SIRT1 in liver after major hepatectomy and IR injury compared to the wild-type group (Figure 7(a)). Similar data were also found in fatty liver (Figure 6(b)). Furthermore, we also detected the levels of succinate dehydrogenase complex iron sulfur subunit B (SDHB) and ATP. The data showed that the expression of SDHB was upregulated in both the N-KO and F-KO groups (Figures 6(a) and 6(b)). More importantly, the knockout of AR increased the content of ATP in both normal and fatty livers after major hepatectomy and IR injury compared to the wild-type group (Figure 7(b)).

## 4. Discussion

Liver regeneration is a highly coordinated process involving complex networks of interactions between various cellular, cytokine, and growth factors [18]. Remnant liver can restore the original hepatic mass after liver surgery through hepatocyte proliferation or oval cell transformation. A variety of cytokines and growth factors have been implicated in regulating liver regeneration [19]. It has been reported that the regeneration capacity of fatty liver was significantly decreased and the risk of postoperative liver failure and graft failure was increased [4, 5, 20]. Our results also confirmed that fatty liver regeneration was delayed in the rat transplantation model and the mouse major hepatectomy and partial IR injury model. Some evidence suggested that impaired fatty liver regeneration maybe due to oxidative stress and metabolism disorder, which resulted in mitochondrial dysfunction and decreased adenosine triphosphate (ATP) production [4, 5, 20]. Therefore, it was worthwhile to clarify the mechanism of fatty liver regeneration and find effective intervention methods to promote fatty liver regeneration.

To our knowledge, this is the first report on the role and mechanism of AR in liver regeneration. In this study, our results demonstrated that the expression of AR was increased in liver after rat liver transplantation and mouse major hepatectomy and IR injury, especially in fatty liver. Furthermore, overexpression of AR in fatty liver was associated with impaired regeneration of fatty liver. The knockout of AR promoted the regeneration of normal and fatty livers in the mouse model. Our data also showed that AR knockout increased the expressions of cyclin A2, B, D1, and E after hepatectomy and IR injury. Cyclins are important downstream effectors of cell cycle and transforming signaling pathways, which play central roles in cell proliferation [21, 22]. Collectively, these data suggested that the inhibition of AR accelerated liver regeneration and could be a potential therapeutic target.

PPARs are nuclear receptor ligand-dependent transcription factors that regulate gene expression, and they are composed of three types: PPAR-*α*, PPAR-*β*, and PPAR-*γ*. Increasing evidence showed that PPARs play important roles in liver generation. A deficiency of PPAR-*α* impaired liver regeneration after partial hepatectomy through regulating gene expression involved in cell cycle control, cytokine signaling, and fat metabolism [23, 24]. There are some papers suggesting that PPAR-*β* was involved in the progression of liver regeneration by regulating PDK1/Akt and E2f signaling-controlled metabolism and proliferation [25, 26]. Moreover, regeneration was impaired in liver-specific PPAR-*γ* null mice with diet-induced hepatic steatosis after hepatectomy. These data suggested that augmenting liver PPAR activity might promote the regeneration of liver after surgery. Recent research showed that AR overexpression caused strong suppression of PPAR-*α* activity by regulating ERK1/2 signaling [27]. In this project, our data showed that the knockout of AR increased the expressions of PPAR-*α* and PPAR-*γ* in both the normal liver and fatty liver groups after major hepatectomy and IR injury compared to the wild-type groups. These data demonstrated that AR negatively regulated the progression of liver regeneration maybe through regulating expressions of PPAR-*α* and PPAR-*γ*.

Mitochondria are significant organelles for hepatocyte energy metabolism. ATP is critical not only for energy supply to maintain cell functions and survival but is also a significant factor in controlling regenerative signaling [28–30]. Decreased ATP production was associated with severe injury, impaired liver regeneration, and increased mortality after transplantation with the small-for-size liver graft [31, 32]. AMP-activated protein kinase (AMPK) is an evolutionarily conserved sensor of cellular energy status that contributes to the restoration of energy homeostasis. AMPK is critical for energy-demanding situations such as liver regeneration by controlling the balance between hepatocyte metabolic adaptation [33]. Deletion of AMPK-*α* delayed liver regeneration after partial hepatectomy by impacting on the G1/S transition phase [34]. PGC1-*α*, as the coactivator of PPAR-*γ*, has been reported to play important roles in mitochondrial biogenesis and energy metabolism. The AR inhibitor increased mitochondrial biogenesis via increasing the expression of Nrf2/HO-1/AMPK/p53 and decreasing the mitochondrial DNA damage in colon cancer cells [35]. In addition, the inhibition of AR also attenuated alcoholic liver disease by activating AMPK and modulating oxidative stress [36]. In this study, our results showed that the knockout of AR increased the expressions of AMPK, SIRT1, PGC1-*α*, SDHB, and ATP content in both normal and fatty livers after major hepatectomy and IR injury. These data indicated that AR inhibition accelerated fatty liver regeneration maybe through regulating mitochondrial biogenesis and energy metabolism. The precise mechanism of AR in regulating energy metabolism during liver regeneration needs to be further studied.

In summary, we demonstrated the role and underlying mechanism of AR in the regeneration of normal and fatty livers after liver surgery. The knockout of AR promoted the regeneration of normal and fatty livers through regulating energy metabolism. The effect of AR in liver regeneration may provide a new potential therapeutic target to accelerate postoperative liver regeneration and decrease the incidence of mobility and mortality.

## Data Availability

The datasets used and/or analyzed during the current study are available from the corresponding author on reasonable request.

## Supplementary Materials

Supplementary Materials Supplementary Table 1: sequences of the primer pairs.
