# Supplementary material for: The Inhibition of Aldose Reductase Accelerates Liver Regeneration through Regulating Energy Metabolism
Source: Oxid Med Cell Longev. 2020 Feb 27;2020:3076131. doi: 10.1155/2020/3076131 (PMC7064854; doi:10.1155/2020/3076131)
Supplement: Supplementary Materials — Supplementary Table 1: sequences of the primer pairs. [file 3076131.f1.docx]

Supplementary Table 1: Sequences of the primer pairs

| Gene name | Sequences |
| --- | --- |
| AR (rat) | Forward: 5’ GTGGACACTTGGACGGCTAT 3’  Reverse: 5’ AGGAGAACCAAGGGGACTGT 3’ |
| PCNA (rat) | Forward: 5’ GCATGGATTCGTCTCACGTC3’  Reverse: 5’ TGGACATGCTGGTGAGGTTC 3’ |
| Cyclin E1 (rat) | Forward: 5’ GGACACAGCTTCGGGTCTGA3’  Reverse: 5’ ATCGGACTGAGAGGTCGGA3’ |
| Cyclin D1 (rat) | Forward: 5’ TCAAGTGTGACCCGGACTG3’  Reverse: 5’ CTTCCCCTTCCTCCTCGGT3’ |
| Cyclin E1 (mouse) | Forward: 5’ GCTGCTAAGGAGGGTGCTAC3’  Reverse: 5’AACCTACAACACCCGAGCAG3’ |
| Cyclin D1 (mouse) | Forward: 5’ GATGGCGATCGTCCTGTCAT 3’  Reverse: 5’ ACAGGCCGCTACAAGAAACA3’ |
| Cyclin B1 (mouse) | Forward: 5’ GAAACATCTGGATGTGCGCC3’  Reverse: 5’ GTTTGGGTCAGCCCCATCAT3’ |
| PPAR-γ (mouse) | Forward: 5’ CATCAGGTTTGGGCGGGAT 3’  Reverse: 5’ CAAATGCTTTGCCAGGGCTC 3’ |
| PPAR-α (mouse) | Forward: 5’ CCCCAGTCTGGTCTTAACCG 3’  Reverse: 5’ GGAACAGACCGCTCAGACTT 3’ |
